# Inhibition of Neutrophil Primary Granule Release during Yersinia pestis Pulmonary Infection

**DOI:** 10.1128/mBio.02759-19

**Published:** 2019-12-10

**Authors:** Kara R. Eichelberger, Grant S. Jones, William E. Goldman

**Affiliations:** a Department of Microbiology and Immunology, University of North Carolina at Chapel Hill, Chapel Hill, North Carolina, USA; Emory University School of Medicine

**Keywords:** *Yersinia pestis*, YopE, YopH, neutrophil degranulation, plague, primary granules, type III secretion

## Abstract

Inhalation of Yersinia pestis causes primary pneumonic plague, the most severe manifestation of plague that is characterized by a dramatic neutrophil influx to the lungs. Neutrophils are ineffective during primary pneumonic plague, failing to control Y. pestis growth in the airways. However, the mechanisms by which Y. pestis resists neutrophil killing are incompletely understood. Here, we show that Y. pestis inhibits neutrophil degranulation, an important line of host innate immune defense. We observed that neutrophils from the lungs of mice infected intranasally with Y. pestis fail to release primary granules throughout the course of disease. Using a type III secretion system (T3SS) injection reporter strain, we determined that Y. pestis directly inhibits neutrophil granule release by a T3SS-dependent mechanism. Combinatorial mutant analysis revealed that a Y. pestis strain lacking both effectors YopE and YopH did not inhibit primary granule release and is killed by neutrophils both *in vivo* and *in vitro*. Similarly, Y. pestis strains injecting only YopE or YopH are able to inhibit the majority of primary granule release from human neutrophils. We determined that YopE and YopH block Rac2 activation and calcium flux, respectively, to inhibit neutrophil primary granule release in isolated human neutrophils. These results demonstrate that Y. pestis coordinates the inhibition of neutrophil primary granule release through the activities of two distinct effectors, and this inhibition promotes Y. pestis survival during primary pneumonic plague.

## Abstract (precis)

Yersinia pestis is the causative agent of plague and is one of the deadliest human pathogens. The pneumonic form of Y. pestis infection has played a critical role in the severity of both historical and modern plague outbreaks, yet the host-pathogen interactions that govern the lethality of Yersinia pestis pulmonary infections are incompletely understood. Here, we report that Yersinia pestis inhibits neutrophil degranulation during infection, rendering neutrophils ineffective and allowing unrestricted growth of Y. pestis in the lungs. This coordinated inhibition of granule release not only demonstrates the pathogenic benefit of “silencing” lung neutrophils but also reveals specific host processes and pathways that could be manipulated to reduce the severity of primary pneumonic plague.

## INTRODUCTION

Neutrophils are rapidly recruited from the bloodstream to the site of infection and thus are among the first immune cells to respond to invading pathogens (1–3). Neutrophils control bacteria using a variety of mechanisms, such as phagocytosis, oxidative burst, neutrophil extracellular traps, and degranulation (4–6). Degranulation is the process by which neutrophils mobilize preformed vesicles called granules to the phagolysosome to kill intracellular bacteria or to the plasma membrane for extracellular release (2, 7). The granules contained within neutrophils can be broadly categorized into four main types: primary, secondary, tertiary, and secretory (8). Of these, primary granules are considered the most proinflammatory and antimicrobial, containing elastase, myeloperoxidase, and a variety of antimicrobial proteins (9). As many of these compounds damage host tissues, primary granules require the strongest stimulus for extracellular release and are deployed as a last defense during severe infections (10–12).

Inhalation of aerosolized droplets containing Yersinia pestis results in primary pneumonic plague, the most lethal manifestation of plague (13, 14). Following inhalation, Y. pestis grows rapidly in the lungs during the early asymptomatic phase of disease (15). Disease then progresses into an acute pneumonia, characterized by severe pulmonary inflammation and a large influx of neutrophils that fail to restrict Y. pestis growth (16, 17). Y. pestis bacteria are closely associated with neutrophils in the lung, yet the consequences of these interactions on the outcome of primary pneumonic plague are unclear.

Y. pestis requires a type III secretion system (T3SS) for pathogenesis. The T3SS can translocate bacterial effectors, called *Yersinia* outer proteins (Yops), through a needle-like apparatus directly into the host cell cytoplasm (18). Y. pestis targets innate immune cells for Yop injection (19). During primary pneumonic plague, neutrophils are the primary target for Y. pestis T3SS injection (17). The Yops are anti-inflammatory and antiphagocytic, with several redundant targets and synergistic effects (18). *Yersinia* utilizes the T3SS to inhibit phagocytosis, oxidative burst, apoptosis, and cytokine production, disarming most of the mechanisms by which neutrophils neutralize bacteria (20–24). However, the ability of Y. pestis to alter neutrophil degranulation during infection has not been explored.

Using a mouse model of primary pneumonic plague developed in our laboratory (25), we report that neutrophils fail to release primary granules in the lungs during infection. We determined that Y. pestis inhibits neutrophil degranulation directly via delivery of T3SS effectors YopE and YopH. During Y. pestis infection, YopE inhibits Rac2 activation and YopH inhibits calcium flux, which are distinct but critical steps in the exocytosis of primary granules from neutrophils. Taken together, the data presented here complete our understanding of the T3SS-mediated mechanisms by which Y. pestis can inhibit neutrophil antimicrobial defenses.

## RESULTS

### Neutrophils fail to release primary granules during primary pneumonic plague.

**FIG 1:**
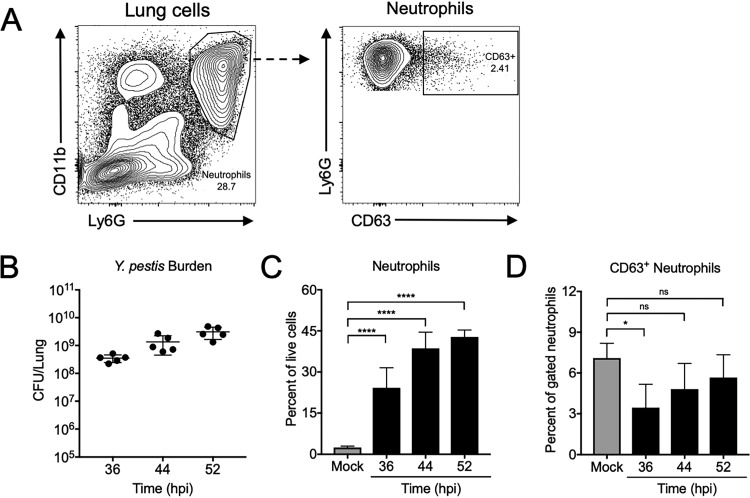
Neutrophils fail to release primary granules during primary pneumonic plague. Mice were inoculated intranasally with 1 × 10^4^ CFU Y. pestis, and lungs were removed and assayed for bacterial burden and neutrophil degranulation at 36, 44, and 52 h postinoculation (hpi). (A) Representative flow cytometry plots showing neutrophils (CD11b^+^ Ly6G^+^) from a mouse lung at 36 h after intranasal inoculation with Y. pestis and the percentage of neutrophils releasing primary granules (CD63^+^). (B) Y. pestis CFU enumerated in the lungs of mice following intranasal inoculation with Y. pestis. (C) Neutrophils recovered from the lungs of mice inoculated intranasally with Y. pestis, expressed as a percentage of total live cells. (D) Percentages of CD63^+^ neutrophils from the lungs of mice inoculated intranasally with Y. pestis. Data shown are pooled from two independent experiments for *n* = 5 mice per group. Bars or lines represent the means, error bars are ±SDs. ns, not significant; *, *P* < 0.05; ****, *P* < 0.001 by one-way ANOVA with Tukey’s multiple correction. Statistics over bars are compared to mock.

To assess lung neutrophil degranulation during primary pneumonic plague, we focused on the release of primary granules, which are released by neutrophils as a last attempt to control bacterial infection (26). Following primary granule exocytosis, membrane-bound CD63 is displayed on the neutrophil surface (27). Thus, the levels of exposed CD63 on neutrophils can be measured as a proxy for primary granule release (9). Mice were inoculated intranasally with 10^4^ CFU Y. pestis (100% lethal dose [LD_100_]), and lungs were harvested at various time points throughout the proinflammatory phase to evaluate primary granule release via the proportion of CD63^+^ neutrophils. A representative flow cytometry plot is shown in Fig. 1A. More than 10^8^ CFU Y. pestis were recovered from the lungs at 36 h postinoculation (hpi), which increased ∼10-fold by 52 hpi (Fig. 1B). Concurrently, we observed a large neutrophil influx, with neutrophils representing 45% of the total lung cells by 52 hpi (Fig. 1C). Despite these proinflammatory conditions, a low percentage of neutrophils released primary granules (CD63^+^), similar to levels of degranulation by neutrophils from mock-infected mice (Fig. 1D). These data indicate that neutrophils recruited to the lungs during primary pneumonic plague do not release primary granules.

### The absence of neutrophil primary granule release is not typical in Gram-negative bacterial pneumonia.

**FIG 2:**
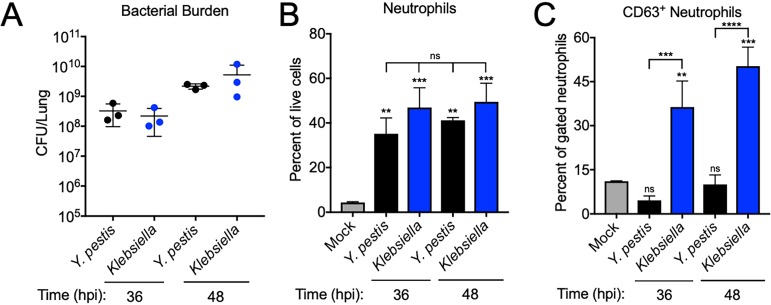
The absence of neutrophil primary granule release is not typical in Gram-negative bacterial pneumonia. Mice were inoculated intranasally with 1 × 10^5^ CFU Klebsiella pneumoniae or 1 × 10^4^ CFU Y. pestis, and lungs were removed and assayed for bacterial burden and neutrophil degranulation at 36 and 48 h postinoculation (hpi). (A) Y. pestis CFU enumerated in the lungs of mice following intranasal inoculation with Y. pestis or K. pneumoniae. (B) Neutrophils recovered from the lungs of mice inoculated intranasally with Y. pestis or K. pneumoniae, expressed as a percentage of total live cells. (C) Percentages of CD63^+^ neutrophils from the lungs of mice inoculated intranasally with Y. pestis or K. pneumoniae. See also Fig. S1 in the supplemental material. Data shown are representative of three independent experiments. Bars or lines represent the means, error bars are ±SDs. **, *P* < 0.01; ***, *P* < 0.005 by one-way ANOVA with Tukey’s multiple correction. Statistics over bars are compared to mock.

Next, we examined if the lack of primary granule release during primary pneumonic plague is a common host response during pulmonary infection. Like Y. pestis, Klebsiella pneumoniae is an extracellular, Gram-negative pathogen that causes acute neutrophilic pneumonia (28). We inoculated groups of mice intranasally with 10^4^ CFU Y. pestis or 10^5^ CFU K. pneumoniae to match disease progression between the two infections for a comparison of lung neutrophil degranulation responses. At both time points analyzed, Y. pestis and K. pneumoniae had similar high bacterial burdens, with ∼5 × 10^8^ CFU in the lungs at 36 hpi that increased approximately 10-fold by 48 hpi (Fig. 2A). Additionally, intranasal inoculation of mice with either Y. pestis or K. pneumoniae resulted in a neutrophil influx to the lungs, with neutrophils comprising 40% to 50% of the total lung cells at both 36 and 48 hpi (Fig. 2B). In contrast, a significantly greater proportion of neutrophils from K. pneumoniae*-*infected mice released primary granules (CD63^+^) than that of the neutrophils from Y. pestis*-* or mock-infected mice (Fig. 2C). This granule release correlates with the amount of elastase (contained within primary granules) in the lung following inoculation with either Y. pestis or K. pneumoniae (see Fig. S1 in the supplemental material). Thus, the lack of primary granule release observed during primary pneumonic plague is not a universal neutrophil response to Gram-negative pathogens during pulmonary infection.

10.1128/mBio.02759-19.1 FIG S1 Neutrophils fail to release primary granules during primary pneumonic plague. Elastase detected by ELISA in the bronchoalveolar lavage fluid (BALF) collected from mice at 36 h after inoculation with 1 × 10^4^ Y. pestis, 1 × 10^5^ K. pneumoniae, or PBS (mock). See Fig. 2. Lines represent the means, error bars are ±SDs. ****, *P* < 0.001 by one-way ANOVA with Tukey’s multiple correction. Download FIG S1, PDF file, 0.1 MB. Copyright © 2019 Eichelberger et al. 2019 Eichelberger et al. This content is distributed under the terms of the Creative Commons Attribution 4.0 International license.

### Y. pestis inhibits neutrophil primary granule release via direct type III secretion system injection.

**FIG 3:**
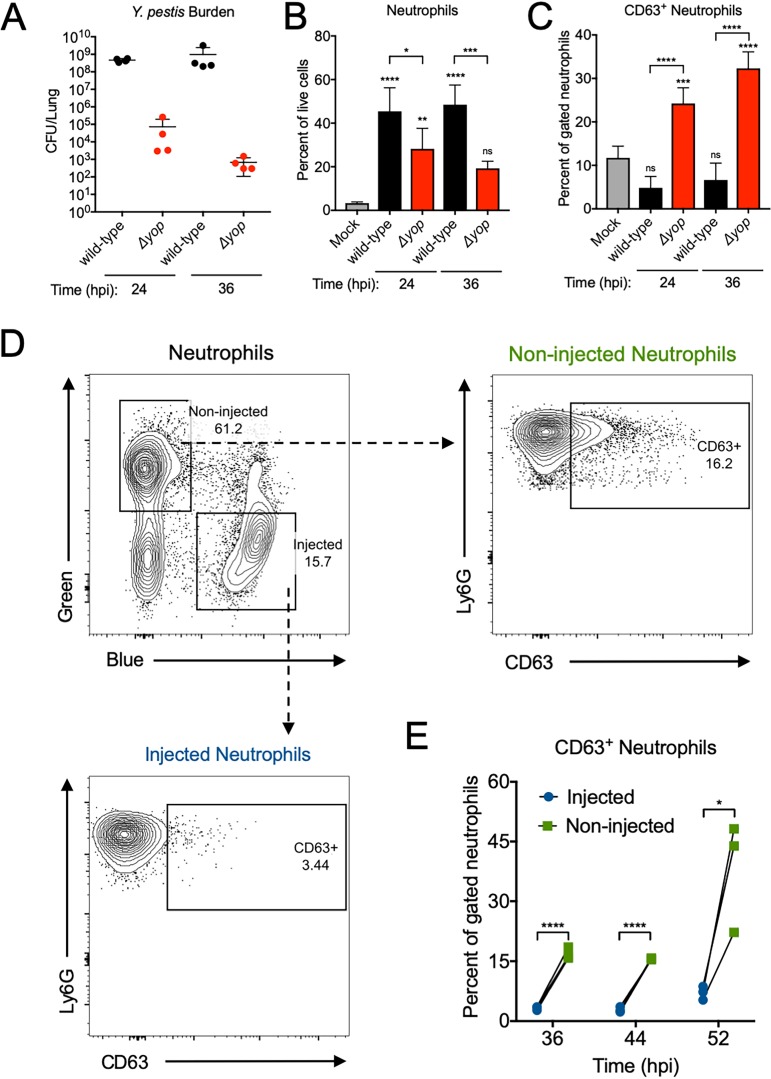
Y. pestis inhibits neutrophil primary granule release via direct type III secretion system injection. Mice were inoculated intranasally with 1 × 10^6^ CFU of the Y. pestis wild-type or Δ*yop* strain, and lungs were removed for analysis of bacterial burden and neutrophil degranulation at 24 h postinoculation (hpi). (A) Y. pestis CFU enumerated in the lungs of mice following intranasal inoculation with the Y. pestis wild-type or Δ*yop* strain. (B) Neutrophils recovered from the lungs of mice inoculated intranasally with the Y. pestis wild-type or Δ*yop* strain expressed as a percentage of total live cells. (C) Percentages of CD63^+^ neutrophils from the lungs of mice inoculated intranasally with the Y. pestis wild-type or Δ*yop* strain. (D) Representative flow plots for neutrophils from a mouse lung 36 h after intranasal inoculation with 1 × 10^4^ CFU Y. pestis YopE-TEM. CCF2-AM staining identifies T3SS-injected (blue) and noninjected (green) neutrophils, and the percent CD63^+^ neutrophils for each population are shown. (E) Percentages of CD63^+^ injected and noninjected neutrophils from the lungs of mice at 36, 44, and 52 h after intranasal inoculation with 1 × 10^4^ CFU Y. pestis YopE-TEM. Lines connect neutrophil populations from the same lung. Data shown are pooled from two independent experiments with *n* = 4 mice per group (A to C) or are representative of two independent experiments (D and E). Bars or lines represent the means, error bars are ±SDs. *, *P* < 0.05; **, *P* < 0.01; ***, *P* < 0.005; ****, *P* < 0.001 by one-way ANOVA with Tukey’s multiple corrections (B and C) or by Student's *t* test (E). Statistics over bars are compared to mock.

Primary granules require strong stimulation for extracellular release following neutrophil activation (10, 29). Thus, the absence of primary granule release could be due to low neutrophil activation or to active inhibition by Y. pestis. We showed previously that neutrophils are the major lung cell target for T3SS injection during primary pneumonic plague (17). To evaluate if T3SS injection inhibits neutrophil primary granule release, we created Y. pestis Δ*yop*, which expresses the T3SS needle apparatus but lacks the seven main Yop effectors (18). We inoculated mice intranasally with 10^6^ CFU of the Y. pestis wild-type or Δ*yop* strain, using a higher inoculum to trigger significant neutrophil recruitment to the lungs for both strains. Y. pestis Δ*yop* failed to proliferate in the lungs, exhibiting a 4-log deficit at 24 hpi and a 6-log deficit at 36 hpi compared to the Y. pestis wild-type CFU in the lungs (Fig. 3A). Additionally, a significantly greater proportion of neutrophils was recruited to the lungs of mice infected with wild-type Y. pestis than to those infected with Y. pestis Δ*yop* at both 24 and 36 hpi (Fig. 3B). However, only neutrophils from the lungs of Y. pestis Δ*yop-*infected mice had significantly higher proportions of primary granule release (CD63^+^) (Fig. 3C). Thus, one or more T3SS effectors inhibit neutrophil primary granule exocytosis during Y. pestis infection.

Next, we hypothesized that T3SS injection inhibits neutrophil degranulation directly. To identify neutrophils injected by the T3SS, we used Y. pestis YopE-TEM and the dye CCF2-AM, which emits green light due to fluorescence resonance energy transfer (FRET). Y. pestis YopE-TEM harbors the wild-type YopE protein plus a β-lactamase moiety fused to the N-terminal 100 amino acids of YopE (17). Infection with this strain leads to the cleavage of CCF2-AM in T3SS-intoxicated neutrophils and disruption of FRET, which changes the dye fluorescence from green to blue (17, 19, 30). This approach allowed us to compare degranulation by noninjected neutrophils (green) or injected neutrophils (blue) in the same lung during Y. pestis infection, as shown in the representative plot in Fig. 3D. Mice were inoculated intranasally with 10^4^ CFU Y. pestis YopE-TEM, and at each time point analyzed, neutrophils injected by the T3SS had significantly less primary granule release (CD63^+^ neutrophils) than noninjected neutrophils from the same mouse lung (Fig. 3E). Collectively, these data demonstrate that Y. pestis directly inhibits neutrophil primary granule release in the lung throughout primary pneumonic plague in a T3SS-dependent manner.

### The type III secretion system effectors YopE and YopH inhibit neutrophil primary granule release.

**FIG 4:**
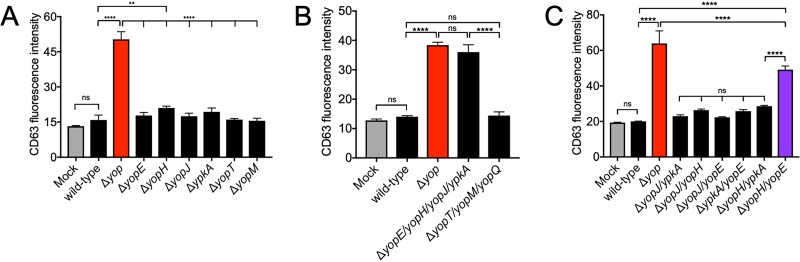
The type III secretion system effectors YopE and YopH inhibit neutrophil primary granule release. CD63 mean fluorescence intensities from purified human peripheral blood neutrophils isolated and inoculated *in vitro* with single Yop deletion (A), combinatorial Yop deletion (B), or double Yop deletion (C) Y. pestis strains. Each strain was tested in triplicates per each experiment, and data shown are representative of three independent experiments. Bars represent means and error bars are ±SDs. **, *P* < 0.01; ****, *P* < 0.001 by one-way ANOVA with Tukey’s multiple corrections.

To determine which T3SS effector(s) inhibited primary granule release, we assessed primary granule release from isolated human neutrophils after infection with various *yop* mutants using our *in vitro* human neutrophil infection and degranulation assay (31). First, we inoculated human neutrophils with Y. pestis wild-type, Δ*yop*, or single *yop* deletion strains for each of the main T3SS effectors: YopE, YopH, YopJ, YpkA, YopT, and YopM (18). One hour after infection, we used flow cytometry to quantify CD63 mean fluorescence intensity (MFI) on the neutrophil surface, which is proportional to the level of primary granule release. Corroborating our *in vivo* results, human neutrophils infected with wild-type Y. pestis had a low CD63 MFI, similar to that of the mock-infected neutrophils. Additionally, the Y. pestis Δ*yop-*infected neutrophils had high levels of primary granule release (Fig. 4A). Neutrophils infected with each of the single *yop* deletion strains had low primary granule release, similar to that of Y. pestis wild-type-infected neutrophils (Fig. 4A). Thus, the loss of a single effector was not sufficient to reverse the T3SS-mediated inhibition of degranulation. This suggests that multiple Yops inhibit primary granule release.

To determine which of the Yops inhibit primary granule release, we designed a combinatorial gene deletion approach and created two strains of Y. pestis, each missing half of the Yop effectors. Human neutrophils infected with Y. pestis lacking YopE, YopH, YopJ, and YpkA had high levels of primary granule release (high CD63 MFI), similar to that of neutrophils infected with Y. pestis Δ*yop* (Fig. 4B). Neutrophils infected with Y. pestis that lacked YopT, YopM, and the regulatory protein YopQ had low levels of primary granule release (low CD63 MFI), similar to that of neutrophils infected with wild-type Y. pestis (Fig. 4B). These data indicate that a combination of YopE, YopH, YopJ, and/or YpkA inhibit neutrophil primary granule release during Y. pestis infection.

Next, we tested if two effectors among YopE, YopH, YopJ, and YpkA were redundantly inhibiting primary granule release. We deleted every combination of two of the four candidate Yops, inoculated human neutrophils with each of these double deletion strains, and then measured primary granule release (CD63 MFI). Only neutrophils infected with Y. pestis Δ*yopEH* had high levels of primary granule release (high CD63 MFI), although these levels were not as high as for Y. pestis Δ*yop*-infected neutrophils (Fig. 4C). Primary granule release was inhibited (low CD63 MFI) in neutrophils infected with all other double Yop deletion strains, similar to that in neutrophils infected with wild-type Y. pestis (Fig. 4C). Because primary granule release was inhibited for each double deletion mutant except the Δ*yopEH* strain, this suggests that YopE and YopH are necessary to inhibit neutrophil primary granule release.

### YopE and YopH independently inhibit primary granule release during primary pneumonic plague.

**FIG 5:**
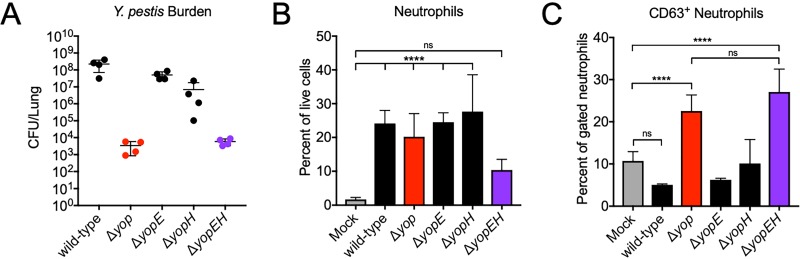
YopE and YopH independently inhibit primary granule release during primary pneumonic plague. Mice were inoculated intranasally with 1 × 10^6^ CFU of the Y. pestis wild-type, Δ*yop*, Δ*yopE*, Δ*yopH*, or Δ*yopEH* strain, and lungs were removed for analysis of bacterial burden and neutrophil degranulation at 24 h postinoculation (hpi). (A) Y. pestis CFU enumerated in the lungs of mice following intranasal inoculation with the Y. pestis wild-type, Δ*yop*, Δ*yopE*, Δ*yopH*, or Δ*yopEH* strain. (B) Neutrophils recovered from the lungs of mice inoculated intranasally with the Y. pestis wild-type, Δ*yop*, Δ*yopE*, Δ*yopH*, or Δ*yopEH* strain, expressed as a percentage of total live cells. (C) Percentages of CD63^+^ neutrophils from the lungs of mice inoculated intranasally with the Y. pestis wild-type, Δ*yop*, Δ*yopE*, Δ*yopH*, or Δ*yopEH* strain. Data shown are pooled from two independent experiments for *n* = 4 mice per group. Bars represent means and error bars are ±SDs. ****, *P* < 0.001 by one-way ANOVA with Tukey’s multiple corrections.

To determine if YopE and YopH inhibit neutrophil primary granule release *in vivo*, we tested YopE- and YopH-deficient Y. pestis mutants in our mouse model of primary pneumonic plague. Based on our *in vitro* experiments with isolated human neutrophils (Fig. 4A to C), we expected that only neutrophils from mice inoculated with Y. pestis Δ*yop* or Δ*yopEH* (which lack both YopE and YopH) would exocytose primary granules. We inoculated mice intranasally with 10^6^ CFU of the Y. pestis wild-type, Δ*yop*, Δ*yopE*, Δ*yopH*, or Δ*yopEH* strain to induce a significant neutrophil influx to the lungs at 24 hpi for comparison of neutrophil granule release among the different infections. As we observed previously, Y. pestis Δ*yop* was impaired for growth in the lungs compared to wild-type Y. pestis at 24 hpi (Fig. 5A). We recovered approximately 10^4^ CFU from the lungs of mice inoculated with Y. pestis Δ*yop* or Δ*yopEH*, an average of 10^7^ CFU from mice inoculated with Y. pestis Δ*yopH*, and around 10^8^ CFU from the lungs of mice inoculated with Y. pestis Δ*yopE* or the Y. pestis wild-type strain (Fig. 5A). The virulence defect in the lungs for Y. pestis Δ*yopEH* suggests the importance of both YopE and YopH for Y. pestis virulence during primary pneumonic plague. Despite differences in bacterial burden, similar proportions of neutrophils (20% to 25% of total lung cells) were recruited to the lungs of mice inoculated with each Y. pestis strain except for Y. pestis Δ*yopEH*, where 10% of total lung cells were neutrophils (Fig. 5B). Consistent with our prediction, only neutrophils from mice inoculated with Y. pestis Δ*yop* or Δ*yopEH* had significant primary granule release (CD63^+^) compared to that of neutrophils from mock-inoculated mice (Fig. 5C). Also, the single deletion Δ*yopE* and Δ*yopH* strains were still able to inhibit neutrophil primary granule release, further supporting the ability of YopE and YopH to independently block neutrophil degranulation.

### Y. pestis inhibits neutrophil Rac2 activation and calcium flux to block primary granule release.

**FIG 6:**
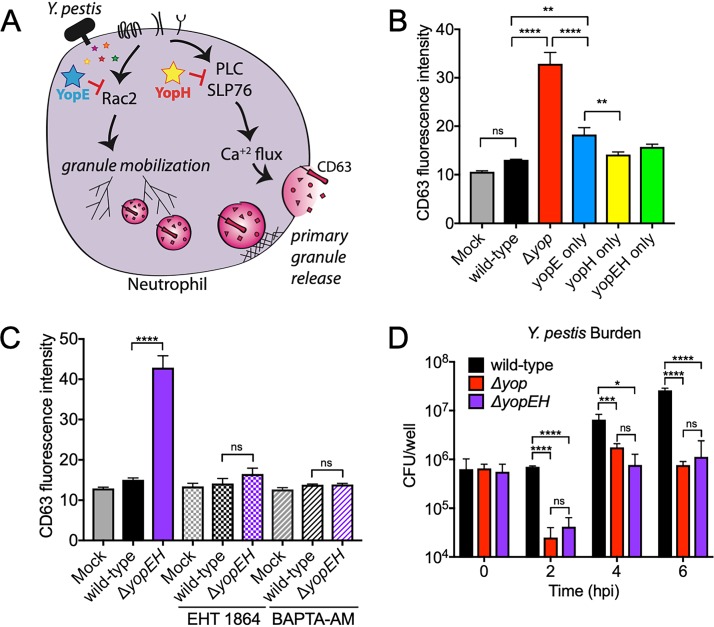
Y. pestis inhibits neutrophil Rac2 activation and calcium flux to block primary granule release. (A) A schematic depicting the pathways involved in neutrophil primary granule release and the host targets of YopE and YopH. (B) CD63 mean fluorescence intensities from isolated human neutrophils measured 1 h after inoculation *in vitro* with the Y. pestis wild-type, Δ*yop*, YopE only, YopH only, or YopEH only strain. (C) CD63 mean fluorescence intensities from isolated human neutrophils preincubated with 50 μM EHT 1864 or 25 μM BAPTA-AM prior to infection with the Y. pestis wild-type or Δ*yopEH* strain. (D) Y. pestis wild-type, Δ*yop*, or Δ*yopEH* CFU recovered from infected human neutrophils at the indicated time points. Each strain or treatment was tested in triplicates per experiment, and data shown are representative of three independent experiments. Bars represent the means, error bars are ±SDs. *, *P* < 0.05; ***, *P* < 0.005; ****, *P* < 0.001 by one-way ANOVA with Tukey’s multiple corrections (B and C) or Student's *t* test (D).

YopE is a GTPase-activating protein that inactivates Rho family GTPases, and Yersinia pseudotuberculosis YopE has been shown to inhibit Rac2 activation in neutrophils (21, 32). YopH is a protein tyrosine phosphatase that inhibits calcium flux by inactivating a variety of host cell kinases, including the PLC/SLP76 signaling axis (33, 34). Both Rac2 activation and Ca^2+^ flux are critical for the release of primary granules from neutrophils. Rac2 activation triggers actin polymerization that directs primary granules to the plasma membrane, where a Ca^2+^ flux is required for fusion of the granules with the plasma membrane during exocytosis (9, 35, 36). Inhibiting just one of these two processes can fully block primary granule release (37, 38). Therefore, we hypothesized that Y. pestis YopE inhibits Rac2 activation and YopH inhibits Ca^2+^ flux to block primary granule release from neutrophils (Fig. 6A). As inhibiting either Rac2 activation or Ca^2+^ flux fully inhibits primary granule release in neutrophils, we predicted that both YopE (inhibiting Rac2 activation that coordinates movement of primary granules to the plasma membrane) and YopH (inhibiting Ca^2+^ flux that allows release of primary granules at the plasma membrane) are sufficient to block primary granule release from neutrophils during Y. pestis infection. We created three strains of Y. pestis deleted for all T3SS effectors except YopE (“YopE only”), YopH (“YopH only”), or both YopE and YopH (“YopEH only”). We infected human neutrophils *in vitro* with each of these strains and measured primary granule release by quantifying levels of cell surface CD63 with flow cytometry. The injection of either YopE or YopH by Y. pestis significantly inhibited neutrophil primary granule release (low CD63 MFI), similarly to Y. pestis strains injecting both YopE and YopH (Fig. 6B). Thus, both YopE and YopH sufficiently inhibit neutrophil primary granule release by targeting separate but critical processes. These data are consistent with our results in which only Y. pestis mutants that lack both YopE and YopH (such as the Δ*yop* or Δ*yopEH* strain) are no longer able to inhibit neutrophil primary granule release.

To test if Rac2 activation and Ca^2+^ flux are targeted by YopE and YopH to inhibit neutrophil primary granule release, we utilized chemical inhibitors for each of these processes to mimic the inhibitory effects of YopE or YopH during Y. pestis infection of neutrophils. Using our *in vitro* human neutrophil infection assay, we pretreated neutrophils with either EHT 1864 (Rac inhibitor, mimicking the effects of YopE) or BAPTA-AM (Ca^2+^ chelator, mimicking the effects of YopH) (34, 39). Then, we infected neutrophils with either the Y. pestis wild-type or Δ*yopEH* strain and quantified primary granule release by measuring cell surface CD63 with flow cytometry. As expected, infection of untreated neutrophils with Y. pestis Δ*yopEH* triggered significant primary granule release (high CD63 MFI), while infection with the Y. pestis wild-type strain inhibited primary granule release from neutrophils (low CD63 MFI) (Fig. 6C). However, primary granule release was inhibited (low CD63 MFI) from Y. pestis Δ*yopEH-*infected neutrophils that were pretreated with either EHT 1864 or BAPTA-AM (Fig. 6C). Thus, chemically inhibiting either Rac2 activation or Ca^2+^ flux in neutrophils can mimic the inhibitory effects of either YopE or YopH, respectively. This provides correlative evidence for our model in which YopE and YopH block the activation of two independent processes required for neutrophil primary granule release (Fig. 6A), and the presence of just one of these Yop effectors is sufficient to inhibit neutrophil degranulation.

During primary pneumonic plague, neutrophils remain in close contact with Y. pestis throughout the proinflammatory phase (25). We hypothesized that YopE- and YopH-mediated inhibition of primary granule release promotes Y. pestis survival, because Y. pestis Δ*yop* and Δ*yopEH* strains (that lack both YopE and YopH) failed to proliferate in the lungs of mice following intranasal inoculation (Fig. 5D). To determine if neutrophils contribute directly to the killing of Y. pestis Δ*yop* or Δ*yopEH*, we inoculated human neutrophils *in vitro* with the Y. pestis wild-type, Δ*yop*, or Δ*yopEH* strain and assayed bacterial survival by enumerating CFU at various time points during infection. Y. pestis wild-type CFU increased by ∼10-fold after 6 h in culture with neutrophils. However, Y. pestis Δ*yop* and Δ*yopEH* CFU were reduced after just 2 h of infection. There was some recovery of CFU at 4 hpi, but both strains failed to proliferate from 4 hpi to 6 hpi (Fig. 6D). These data demonstrate the necessity of YopE and YopH in promoting Y. pestis survival during initial interactions with neutrophils.

## DISCUSSION

A hallmark of primary pneumonic plague is massive neutrophil recruitment to the lung during the proinflammatory phase of disease. However, these neutrophils are ineffective at controlling Yersinia pestis growth in the lung or dissemination to the bloodstream. Y. pestis can inhibit neutrophil phagocytosis, oxidative burst, and apoptosis via type III secretion system (T3SS) injection, but the effects of this pathogen’s T3SS on neutrophil degranulation have not been explored. Our study highlights an additional Y. pestis virulence mechanism in which T3SS injection of neutrophils inhibits degranulation to promote survival during primary pneumonic plague. We demonstrated that Y. pestis directly inhibits the release of primary granules, the most proinflammatory and antimicrobial granule type. Through combinatorial T3SS effector mutant analysis, we identified that Y. pestis*-*mediated inhibition of neutrophil primary granule release is dependent on the delivery of T3SS effectors YopE and YopH. By using neutrophils isolated from the lungs of mice inoculated intranasally or neutrophils isolated from human blood and inoculated *in vitro*, we showed that primary granules are released only when both YopE and YopH are absent during Y. pestis infection.

Consistent with these data, we also showed that YopE and YopH are both sufficient to independently inhibit neutrophil primary granule release. During Y. pseudotuberculosis infection of neutrophils, YopE inactivates the Rho GTPase Rac2 and YopH inhibits Ca^2+^ flux (21, 34). Rac2 activity and Ca^2+^ flux tightly regulate the mobilization and exocytosis of primary granules from neutrophils (37, 38), and our experiments reveal that chemical inhibition of either Rac2 activation or Ca^2+^ flux is sufficient to inhibit neutrophil primary granule release during Y. pestis Δ*yopEH* infection. Thus, our data support a model in which Y. pestis inhibits neutrophil primary granule release via two independent mechanisms: YopE-mediated inhibition of Rac2 activation, thereby preventing primary granule mobilization to the plasma membrane, and YopH-mediated inhibition of Ca^2+^ flux, preventing the exocytosis of primary granules at the plasma membrane (Fig. 6A).

While injection of either YopE or YopH inhibits neutrophil primary granule exocytosis, neutrophils do not release primary granules during Y. pestis Δ*yopEH* infection to the same high level as neutrophils during Y. pestis Δ*yop* infection (Fig. 4C). This suggests a minor role for additional effectors in targeting other steps in the exocytosis of primary granules. In support of this, we observe that neutrophils infected with Y. pestis Δ*yopEHJ ypkA* release primary granules to the same extent as neutrophils infected with Y. pestis Δ*yop* (Fig. 4B). Therefore, YpkA or YopJ may play a small role in primary granule release inhibition that is mediated largely by YopE and YopH. YpkA disrupts the actin cytoskeleton and YopJ inhibits mitogen-activated protein kinase (MAPK) signaling via acetyltransferase activity, and both actin dynamics and MAPK have been implicated in regulating neutrophil degranulation (40–43). However, our data reveal that the main Y. pestis effectors responsible for the inhibition of primary granule release are YopE and YopH.

Recently, it was demonstrated that Y. pseudotuberculosis inhibits release of secondary granules from neutrophils *in vitro* (44). YopE and YopH are cooperatively implicated in this process, as neutrophil secondary granule release is partially inhibited during *in vitro* infections with Y. pseudotuberculosis Δ*yopE* or Δ*yopH* single deletion mutants (44). In contrast, we show that Y. pestis YopE and YopH inhibit degranulation via independent and nonsynergistic mechanisms. Primary granules are released only during *in vitro* and *in vivo* infection with Y. pestis strains deleted for both YopE and YopH. We also report that neutrophils infected with either Y. pestis Δ*yopE* or Δ*yopH* are still fully inhibited for primary granule release, similar to that for neutrophils infected with the Y. pestis wild-type strain. This discrepancy—synergistic versus independent inhibition of neutrophil degranulation by YopE and YopH during infection—may be due to differences between either the interactions of neutrophils with the different *Yersinia* species or the control of exocytosis of the different granule types (45, 46).

Inhibition of neutrophil primary granule release by YopE and YopH is critical for the growth of Y. pestis during primary pneumonic plague (Fig. 5A). This is consistent with our *in vitro* human neutrophil infection assay, in which Y. pestis Δ*yop* and Δ*yopEH* strains are killed within the first 2 h after inoculation (Fig. 6D). The enhanced killing of Y. pestis strains lacking YopE and YopH may also be due to enhanced phagocytosis, as both YopE and YopH play a role in the T3SS-mediated inhibition of neutrophil phagocytosis. Regardless, these results indicate that the inhibition of neutrophil function may promote Y. pestis survival as the bacteria come in contact with these cells. In addition to promoting Y. pestis survival, T3SS-mediated inhibition of primary granule release prevents the release of proinflammatory mediators that could damage lung tissue but also trigger a productive immune response (47). Indeed, normal lung architecture is preserved as neutrophils begin to accumulate in the lung (25). However, significant lung damage and destruction of the alveoli occurs at very late time points, preceding the death of the host. If Y. pestis fully inhibits neutrophil degranulation, then what contributes to this late damage? Our data suggest that the non-T3SS-injected neutrophils may be driving some of the end-stage lung damage.

As the proinflammatory phase of primary pneumonic plague progresses, neutrophils continue to accumulate in the alveolar spaces (25). There is also a dramatic increase in the levels of proinflammatory cytokines in the lung throughout the proinflammatory phase of disease (25, 48). As the infection progresses further, fresh neutrophils called to the lung may be rapidly activated by the highly inflammatory lung environment, triggering degranulation of damaging proteases before Y. pestis can deliver T3SS effectors to block degranulation. Our data using the T3SS reporter strain Y. pestis YopE-TEM showed high levels of degranulation (CD63^+^) from noninjected neutrophils at the latest time point analyzed (52 hpi) (Fig. 3D). These activated degranulating neutrophils could be driving the severe host tissue damage and further contribute to the acute pneumonia that ultimately kills the host.

Taken together, our data highlight a virulence strategy in which Y. pestis T3SS injection inhibits neutrophil function to disrupt the balance of neutrophil responses, rendering them ineffective: Y. pestis inhibits neutrophil degranulation following initial contact with the bacteria, and then excessive degranulation occurs later when Y. pestis burdens are beyond control. In support of this, altering the numbers of neutrophils in the lung during Y. pestis infection can overcome the T3SS-mediated disruption to neutrophil responses and improve disease outcomes. Inducing early recruitment of neutrophils to the lungs during primary pneumonic plague enhances control of Y. pestis growth and reduces the severity of disease (49). Taking the opposite approach and depleting neutrophils has no effect on Y. pestis growth but significantly reduces late-stage host damage and disease severity, presumably because there are fewer neutrophils present in the lungs to degranulate during the final stages of infection (17). Thus, a balance of Y. pestis to the number of functional neutrophils in the lung plays a critical role in determining the outcome of infection, and inhibiting granule release may be important for Y. pestis to skew that balance in favor of severe pneumonia and increased virulence.

## MATERIALS AND METHODS

### Bacterial strains and growth conditions.

Strains used in this study are detailed in Table S1 in the supplemental material. The fully virulent Yersinia pestis strain CO92 was obtained from the U.S. Army, Fort Detrick, Frederick, MD. The presence of pCD1 and the *pgm* locus was confirmed for each Y. pestis strain by PCR before use. Y. pestis was grown on brain heart infusion (BHI) agar (Difco Laboratories) at 26°C for 2 days. KPPR1S, a streptomycin- and rifampin-resistant mutant of K. pneumoniae ATCC 43816, was grown on LB agar with 30 μg/ml rifampin at 37°C for 1 day.

10.1128/mBio.02759-19.2 TABLE S1 Bacterial strains and primers used in this work. Download Table S1, PDF file, 0.1 MB. Copyright © 2019 Eichelberger et al. 2019 Eichelberger et al. This content is distributed under the terms of the Creative Commons Attribution 4.0 International license.

### Animals and animal infections.

Naïve 6-to-8-week-old female C57BL/6J mice were obtained from Jackson Laboratories and housed in animal biosafety level 3 facilities at University of North Carolina at Chapel Hill prior to inoculation. All experiments involving mice were reviewed and approved by the Institutional Animal Care and Use Committee at UNC Chapel Hill under protocol 17-258. Y. pestis CO92 growth from a BHI agar plate was used to start liquid cultures in 2 ml BHI broth grown for 12 h at 26°C. Cultures were then diluted 1:200 in 10 ml BHI supplemented with 2.5 mM CaCl_2_ and grown for 12 to 16 h at 37°C with constant shaking at 250 rpm. K. pneumoniae VK148 was grown in 2 ml LB broth for 12 to 16 h at 37°C. Mice were lightly anesthetized with 50 to 100 mg/kg of body weight ketamine and 5 to 10 mg/kg xylazine and then inoculated intranasally with a lethal dose of bacteria suspended in 20 μl sterile phosphate-buffered saline (PBS). A dose of either 1 × 10^4^ or 1 × 10^6^ CFU was used for Y. pestis, and 1 × 10^5^ CFU was used for K. pneumoniae. Mice in the mock-inoculated groups were inoculated with 20 μl sterile PBS. Following infection, mice were monitored every 12 h until euthanasia at the designated time points.

### Generation of lung single-cell suspensions for flow cytometry.

Mice were euthanized with a lethal dose of sodium pentobarbital (Socumb 6G, 150 mg/kg). Lungs were inflated via tracheal cannulation with 1 ml digestion buffer: RPMI (Gibco) with 2 mg/ml collagenase IV (Gibco), 3 mM CaCl_2_, 5 U/ml DNase (Thermo Scientific), 5 U/ml dispase II (Corning), 5% fetal bovine serum (Sigma), and 10 mM HEPES buffer (Affymetrix). Lungs were excised from the mouse, placed in 5 ml digestion buffer, and incubated for 45 min at 37°C with 5% CO_2_ with periodic agitation. Room temperature PBS was then added to each digestion to bring the volume to 30 ml, and samples were vortexed to create a single-cell suspension. For determination of bacterial burden, a portion of this lung cell suspension was removed, and serial dilutions were plated on BHI agar (for Y. pestis) or LB agar with 30 μg/ml rifampin (for K. pneumoniae) and reported as CFU/lung. The remaining cell suspension was filtered through a 70-μm filter and centrifuged at 700 × *g* for 5 min at 4°C. The cell pellet was resuspended in 2 ml ACK buffer (150 mM NH_4_Cl, 10 mM KHCO_3_, 0.1 mM Na_2_EDTA) for 2 min to lyse erythrocytes. Eighteen milliliters of ice-cold flow buffer (PBS with 5% fetal bovine serum [FBS], 2 mM EDTA, 0.1% sodium azide) was added to each sample, and samples were filtered through 70-μm filters. Lung single-cell suspensions were kept on ice until antibody staining.

### Antibody staining for flow cytometry.

Three milliliters of each lung single-cell suspension was removed and centrifuged at 700 × *g* for 5 min at 4°C. The cells were washed once in flow buffer and then stained in a 100-μl volume with Zombie Red fixable viability dye (BioLegend) for 15 min at room temperature. Cells were washed once in flow buffer and resuspended in 100 μl flow buffer. Fc Block (BioLegend) was performed on ice for 5 min followed by antibody staining with phycoerythrin-conjugated CD11b (CD11b-PE; BioLegend), Brilliant Violet 510-conjugated Ly6G (Ly6G-BV510; BioLegend), and allophycocyanin-conjugated CD63 (CD63-APC; Invitrogen) for an additional 30 min on ice. Cells were washed twice with flow buffer and resuspended in 500 μl of 2% formalin in flow buffer and incubated for 20 min at room temperature for fixation. Samples were washed once and resuspended in flow buffer with gentamicin (100 μg/ml) for removal from the biosafety level 3 laboratory in accordance with CDC and UNC biosafety guidelines. Cells were acquired on an Attune NxT cytometer and analyzed using FlowJo v10.4.2 software. Gating was performed to exclude debris and doublets, and neutrophil populations were identified as Zombie Red^−^ CD11b^+^ Ly6G^+^. CD63^+^ neutrophils were gated based on a CD63 fluorescence minus one staining control sample, which contains all antibodies in the assay except CD63-APC.

### Antibody staining with CCF2-AM for flow cytometry.

Three milliliters of each lung single cell suspension was removed and centrifuged at 700 × *g* for 5 min at 4°C. The cells were washed once in flow buffer and then stained in a 100-μl volume with Zombie Red fixable viability dye for 15 min at room temperature. Cells were washed once in flow buffer and resuspended in 100 μl flow buffer. Fc Block was performed on ice for 5 min followed by antibody staining with CD11b-PE, Ly6G-APC/Fire 750 (BioLegend), and CD63-APC for an additional 30 min on ice. Cells were washed twice with flow buffer and resuspended in 100 μl of flow buffer. Twenty microliters of 6× CCF2-AM (β-lactamase loading solution; Invitrogen) was added to each sample, and samples were incubated for 30 min in the dark at room temperature. CCF2-AM is a cell-permeable dye with coumarin conjugated to fluorescein by a cephalosporin linker. Due to fluorescence resonance energy transfer (FRET), the CCF2-AM dye fluoresces green (520 nm) following excitation with a 405-nm laser. Upon cleavage by β-lactamase, the FRET activity is lost and coumarin emits blue fluorescence (477 nm) following excitation at 405 nm. Therefore, we can differentiate neutrophils that were injected by the T3SS from those that were not by determining the amount of green or blue fluorescence emitted by neutrophils (17). Samples were washed once with flow buffer, resuspended in 500 μl of 2% formalin in flow buffer, and incubated for 20 min at room temperature for fixation. Samples were washed once with flow buffer and resuspended in flow buffer with gentamicin (100 μg/ml) for removal from the biosafety level 3 laboratory in accordance with CDC and UNC biosafety guidelines. Cells were acquired on an Attune NxT cytometer and analyzed using FlowJo software. Gating was performed to exclude debris and doublets and neutrophil populations were identified as Zombie Red^−^ CD11b^+^ Ly6G^+^. Noninjected neutrophil populations were identified as blue^−^ green^+^ and injected neutrophil populations were blue^+^ green^−^ using the 405-nm laser. CD63^+^ neutrophils were gated based on CD63 fluorescence minus one staining control sample, which contains all antibodies in the assay except CD63-APC.

### Elastase protein analysis.

Mice were euthanized with a lethal dose of sodium pentobarbital (Socumb 6G, 150 mg/kg). Lungs were inflated via tracheal cannulation with 1 ml ice-cold PBS with protease inhibitors (protease inhibitor cocktail III; Millipore), and the liquid was retracted to collect bronchoalveolar lavage fluid (BALF). A total of 3 ml BALF was collected from each mouse. One milliliter of each sample was centrifuged at 21,000 × *g* for 5 min at 4°C. The supernatant was drawn into a 1-ml syringe and filtered through a 0.22-μm filter to remove bacteria. The centrifugation and filtration steps were repeated, and a portion of the sample was plated on BHI agar to verify the removal of Y. pestis. Samples were removed from the biosafety level 3 laboratory in accordance with CDC and UNC biosafety guidelines. Dilutions of each BALF sample were made and analyzed for the presence of elastase using a DuoSet Mouse Neutrophil Elastase/ELA2 enzyme-linked immunosorbent assay (ELISA) kit (R&D systems) as specified by the manufacturer’s protocol.

### Mutations in Y. pestis pCD1 plasmid.

The strains created and primers used in this study are detailed in Table S1. The deletion of Yop proteins from the pCD1 plasmid of Y. pestis was made either using a modified form of lambda Red recombination or by homologous recombination with pSR47S. For lambda Red recombination, 500-bp upstream and 500-bp downstream sequences of the desired region for deletion were amplified by PCR and combined in splicing by overhang extension (SOE) PCR with a Kan^r^ cassette flanked by FLP recombination target (FRT) sites for allelic replacement of the wild-type open reading frame (ORF) (50). The products were transformed into Y. pestis strains harboring pWL204, a plasmid containing the recombinase genes (51). Following successful recombination, the Kan^r^ cassette was resolved by the introduction of pSkippy, a Tet^s^ derivative of pFLP3 harboring an Amp^r^ cassette and *sacB* and carrying the FLP recombinase gene under the control of the *lac* promoter (52).

To delete YopH, YopJ, and YpkA genes (KO1 region of the pCD1 plasmid), homologous recombination with pSR47S was used (53). The upstream and downstream sequences of this region were amplified by PCR using oligonucleotides KO1 upstream F, KO1 upstream R, KO1 downstream F, and KO1 downstream R. The PCR products were digested with SalI and NotI and subsequently ligated into pSR47S. An Escherichia coli *pir*-containing strain (S17) harbored the subsequent plasmid, creating strain KE52. A mating was performed in which equal volumes of E. coli and Y. pestis were mixed, plated on BHI agar, and allowed to incubate at 26°C overnight. The resulting lawn of cells was scraped into 1 ml PBS, and serial dilutions were plated on BHI agar containing polymyxin B (25 μg/ml) to select against E. coli and kanamycin (50 μg/ml) to select against Y. pestis lacking the plasmid. Transconjugants were streaked on BHI agar containing 5% sucrose to select for colonies that had undergone a second recombination step and lost the vector. Colonies were screened for kanamycin sensitivity and an in-frame deletion was confirmed using PCR.

Construction of each of the Y. pestis *yop* mutants generated in this study is detailed in Table S2.

10.1128/mBio.02759-19.3 TABLE S2 Construction of Y. pestis Yop deletion strains and the oligonucleotides used for lambda Red recombination to generate the strains used in this study. Download Table S2, PDF file, 0.1 MB. Copyright © 2019 Eichelberger et al. 2019 Eichelberger et al. This content is distributed under the terms of the Creative Commons Attribution 4.0 International license.

### Human neutrophil isolation.

Human peripheral blood samples (30 ml) were obtained from anonymous consenting donors, representing a 50:50 ratio of male/female donors. Peripheral blood was collected into EDTA-containing vacuum tubes by standard venipuncture at the UNC Center for AIDS Research HIV/STD Laboratory Core under IRB protocols 96-0859 and 08-0328. Within 20 min of collection, blood was mixed with 20 ml of a 3% dextran-0.9% sodium chloride solution and incubated at room temperature for 20 min for erythrocyte sedimentation. The top serum layer was removed and centrifuged at 250 × *g* at 20°C for 10 min. The pellet was resuspended in 25 ml 0.9% sodium chloride and underlaid with 10 ml Ficoll-Paque Plus (GE Healthcare). The gradient was centrifuged at 400 × *g* for 40 min at 20°C. All layers were removed, leaving the neutrophil and erythrocyte pellet. To lyse erythrocytes, the pellet was resuspended in 20 ml 0.2% sodium chloride solution and incubated at room temperature for 30 s, and tonicity was restored with the addition of 20 ml 1.6% sodium chloride solution. The suspension was centrifuged at 250 × *g* at 20°C for 6 min, and the erythrocyte lysis process was repeated. The final pellet was resuspended in 1 ml PBS, and neutrophils were enumerated using a hemocytometer slide. The neutrophils were resuspended at a final concentration of 2 × 10^6^ neutrophils/ml in neutrophil medium: RPMI 1640 without phenol red (Gibco), buffered to pH 7.2 with 0.25 mM HEPES and supplemented before use with 5% FBS, 2 mM l-glutamine, and 2.5 mM CaCl_2_. Neutrophils were used immediately after isolation.

### Treatment of neutrophils with inhibitors.

BAPTA-AM (Life Technologies) and EHT 1864 (Cayman Chemical Company) were reconstituted in dimethyl sulfoxide (DMSO) and added to the designated neutrophil wells for final concentrations of 50 μM (EHT 1864) and 25 μM (BAPTA-AM) after allowing the freshly isolated neutrophils to rest at 37°C for 30 min. After adding the inhibitors, neutrophils were incubated at 37°C for an additional 30 min prior to inoculation with Y. pestis.

### Infection of human neutrophils.

Twenty-four-well tissue culture plates were preincubated with FBS for 1 h at 37°C and washed twice with PBS prior to seeding with 500 μl of isolated neutrophils for a concentration of 1 × 10^6^ neutrophils/well. The seeded neutrophils were allowed to rest at 37°C for 30 min prior to inoculation. Prior to neutrophil isolation, Y. pestis CO92 growth from a BHI agar plate was used to start liquid cultures in BHI broth grown for 12 h at 26°C. Cultures were then diluted to an optical density at 620 nm (OD_620_) of 0.2 in BHI broth supplemented with 2.5 mM CaCl_2_ and grown for 5 h at 37°C with constant shaking at 250 rpm. Each tissue culture well was inoculated with 1 × 10^6^ CFU Y. pestis diluted in 20 μl of PBS for a multiplicity of infection (MOI) of 1. Neutrophils in the mock groups were inoculated with 20 μl of PBS. Triplicate wells were inoculated for each strain tested. After inoculation, neutrophils were incubated at 37°C for 1 h while rocking before analysis with flow cytometry.

### Flow cytometry of human neutrophils.

After infection, the well contents were collected and centrifuged at 1,000 × *g* for 2 min. The cell pellet was washed twice with flow buffer (2% FBS in PBS) and resuspended in 50 μl of flow buffer for staining. Neutrophils were stained for 25 min with CD63-PE-Cy7 (Invitrogen) on ice. Neutrophils were washed once in flow buffer and resuspended in flow buffer containing propidium iodide (Invitrogen) to exclude dead cells. Neutrophils were acquired using a Millipore Guava 6HT flow cytometer and analyzed with the InCyte EasyCyte v3.1 software.

### Bacterial survival assays.

Isolated human neutrophils were inoculated as described above at an MOI of 1. Following inoculation, tissue culture plates were spun at 700 × *g* for 10 min at 12°C. Medium was aspirated and replaced with fresh neutrophil medium, and plates were incubated at 37°C. At each time point, the medium was aspirated, and 100 μl of 5% saponin (diluted in PBS) was added to each well to lyse neutrophils. Neutrophils were kept at 37°C for 10 min. Next, 900 μl of BHI broth was added to each well, and serial dilutions were plated on BHI agar to enumerate CFU. Control wells containing neutrophil medium without neutrophils were also inoculated with Y. pestis, and a portion was removed for serial dilutions plated on BHI agar at each time point to serve as a medium-only control.

### Statistical analyses.

Details for statistical analyses such as replicates, group numbers, the statistical test used, and the definition of statistical significance are given in the figure legends for each experiment. Analysis of mouse lung flow cytometry data was performed with FlowJo version 10.4.2, and analysis of human neutrophil flow cytometry data was performed with Millipore InCyte-Analysis version 3.1. Data were graphed and analyzed for statistical significance in Prism 7.0b. Data were analyzed by one-way analysis of variance (ANOVA) with Tukey’s multiple-comparison test or by Student's *t* test and are presented as the means with standard deviations (SDs).
