# Supplementary material for: Inhibition of Neutrophil Primary Granule Release during Yersinia pestis Pulmonary Infection
Source: mBio. 2019 Dec 10;10(6):e02759-19. doi: 10.1128/mBio.02759-19 (PMC6904878; doi:10.1128/mBio.02759-19)
Supplement: FIG S1 [file mBio.02759-19-sf001.pdf]

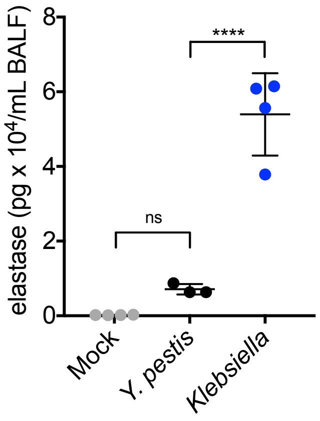

Figure S1. Neutrophils fail to release primary granules during primary pneumonic plague. Elastase detected by ELISA in the bronchoalveolar lavage fluid (BALF) collected from mice at 36 hours after inoculation with 1x10^4 *Y. pestis,* 1x10^5 *K. pneumoniae,* or PBS (mock). See **Figure 2**. Lines represent the mean, error bars are ± SD and ****p<0.001 by one-way ANOVA with Tukey's multiple correction.
