# Supplementary material for: Inhibition of Neutrophil Primary Granule Release during Yersinia pestis Pulmonary Infection
Source: mBio. 2019 Dec 10;10(6):e02759-19. doi: 10.1128/mBio.02759-19 (PMC6904878; doi:10.1128/mBio.02759-19)
Supplement: TABLE S1 [file mBio.02759-19-st001.pdf]

Table S1. Bacterial strains and primers used in this work.

Number	Description/Sequence	Source/Reference
Strain number	*Strain description/genotype*	*Source*
YP03.5	*Yersinia pestis* CO92, wild-type *(pCD1$^+$, pMT1$^+$, pPCP1$^+$, pgm$^+$)*	1
YP489	*Y. pestis* CO92 *Δyop [ΔyopE ΔKO1 (yopH, ypkA, yopJ), ΔKO2 (yopT, yopM, yopQ)]*	This study
YP266-1	*Y. pestis* CO92 *ΔyopE*	2
YP373-1	*Y. pestis* CO92 *ΔyopH*	3
YP100	*Y. pestis* CO92 *ΔyopJ*	2
YP268-1	*Y. pestis* CO92 *ΔyopT*	2
YP267-1	*Y. pestis* CO92 *ΔyopM*	2
YP269-1	*Y. pestis* CO92 *ΔypkA*	2
YP487-1	*Y. pestis* CO92 *ΔyopE/yopH/yopJ/ypkA*	This study
YP528	*Y. pestis* CO92 *ΔyopT/yopM/yopQ*	This study
YP493	*Y. pestis* CO92 *ΔyopE/yopH*	This study
YP529	*Y. pestis* CO92 *ΔyopE/yopJ*	This study
YP500-2	*Y. pestis* CO92 *ΔyopE/ypkA*	This study
YP526	*Y. pestis* CO92 *ΔyopH/yopJ*	This study
YP525	*Y. pestis* CO92 *ΔyopH/ypkA*	This study
YP522	*Y. pestis* CO92 *ΔypkA/yopJ*	This study
YP520	*Y. pestis* CO92 yopE only [*ΔKO1 (yopH, ypkA, yopJ) ΔKO2 (yopT, yopM, yopQ)*]	This study
YP517-1	*Y. pestis* CO92 yopH only [*ΔyopE ΔKO2 (yopT, yopM, yopQ) ypkA-yopJ::kan*]	This study
YP533-2	*Y. pestis* CO92 yopEH only [*ΔKO2 (yopT, yopM, yopQ) ΔypkA-yopJ*]	This study
YPI134-1	*Y. pestis* CO92 YopE-TEM	4
VK148	*Klebsiella pneumoniae* KPPR1S, *Rif^R and Str^R derivative of ATCC 43816*	5
KE52	*Escherichia coli* S17 pSR47S-KO1	This study
Primer number	*Primer name and Sequence*	*Source*
BG2930	yopE up F, 5'-TAGTGAGTTATTATCCAGGCTGTTC-3'	This study
BG2931	yopE up R + P1, 5'-GAAGCAGCTCCAGCCTACACCATGACTATTTATTACCTTGGCTATTA-3'	This study
BG2932	yopE down F + P4, 5'-GGTCGACGGATCCCCGGAATTGATATGGATAAAAACAAGGGGATAG-3'	This study
BG2933	yopE down R, 5'-ATGTACCTGTGAGCCATCGTTTAAT-3'	This study
BG2971	KO1 upstream F, 5'- GTCGACAATATGCCACCCGTTATCTATG-3'	This study
BG2972	KO1 upstream R, 5'-GGATCCCACCACTTTGCCAATCAAAGAAAC-3'	This study
BG2973	KO1 downstream F, 5'-GGATCCCATGCTTTACTCATCCCCAT-3'	This study
BG2974	KO1 downstream R, 5'-GCGGCCGCATTCTGGGACGGGTTTAATC-3'	This study
BG2977	KO2 upstream F, 5'-GTCGACAACAGGGCATGGCACCTCCC-3'	This study
BG2989	KO2 upstream R + P1, 5'-GAAGCAGCTCCAGCCTACACGGATGAAGCTATATTAAAGAG-3'	This study
BG2990	KO2 downstream F + P4, 5'-GGTCGACGGATCCCCGGAATCATATTGAATGCCTTTCTG-3'	This study
BG2978	KO2 downstream R, 5'-GCGGCCGCCCCTGACGGTTTCAAAA-3'	This study
BG3006	KO1 site R, 5'-GCCCATAGACTCCTCCTATGCTTAAG-3'	This study
BG3011	yopH up F, 5'-TCGCACGGAAAACTGCATCC-3'	This study
BG3012	yopH up R + P1, 5'-GAAGCAGCTCCAGCCTACACCATGCTTCCCTCCTTAATTAA-3'	This study
BG3013	yopH down F + P4, 5'-GGTCGACGGATCCCCGGAATTAATGTAAATATTTATTCCT-3'	This study
BG3014	yopH down R, 5'-GGCGGTGGTACTAAAAATAGG-3'	This study
BG3019	yopJ up F, 5'-TTGCGAGAGCTGACGACCATC-3'	This study
BG3020	yopJ up R + P1, 5'-GAAGCAGCTCCAGCCTACACCATTTATTTATCCTTATTCAGGG-3'	This study
BG3021	yopJ down F + P4, 5'-GGTCGACGGATCCCCGGAATTAATGTATTTTGGAAATCTTGCTCC-3'	This study
BG3022	yopJ down R, 5'-CTGGGTATCGGTGCTATGATCG-3'	This study
BG3043	ypkA up F, 5'-CCATCCGATATATCAGTTTCCAACTG-3'	This study
BG3044	ypkA up R + P1, 5'-CCATCCGATATATCAGTTTCCAACTG-3'	This study
BG3045	ypkA down F + P4, 5'-GGTCGACGGATCCCCGGAATCATGCTTTACTCATCCCC-3'	This study
BG3065	ypkA-yopJ up F + P1, 5'-GAAGCAGCTCCAGCCTACACCATGCTTTACTCATCCCC-3'	This study

Supplementary References

1. **Doll JM**, **Zeitz PS**, **Ettestad P**, **Bucholtz AL**, **Davis T**, **Gage K**. 1994. Cat-transmitted fatal pneumonic plague in a person who traveled from Colorado to Arizona. Am J Trop Med Hyg **51**:109–114.

2. **Stasulli NM**, **Eichelberger KR**, **Price PA**, **Pechous RD**, **Montgomery SA**, **Parker JS**, **Goldman WE**. 2015. Spatially Distinct Neutrophil Responses within the Inflammatory Lesions of Pneumonic Plague. mBio **6**:e01530–15.

3. **Price PA**, **Jin J**, **Goldman WE**. 2012. Pulmonary infection by *Yersinia pestis* rapidly establishes a permissive environment for microbial proliferation. Proc Natl Acad Sci U S A **109**:3083–3088.

4. **Pechous RD**, **Sivaraman V**, **Price PA**, **Stasulli NM**, **Goldman WE**. 2013. Early Host Cell Targets of *Yersinia pestis* during Primary Pneumonic Plague. PLoS Pathog **9**:e1003679.

5. **Palacios M**, **Broberg CA**, **Walker KA**, **Miller VL**. 2017. A Serendipitous Mutation Reveals the Severe Virulence Defect of a *Klebsiella pneumoniae fepB* Mutant. mSphere **2**:e00341-17.
