# Supplementary material for: Inhibition of Neutrophil Primary Granule Release during Yersinia pestis Pulmonary Infection
Source: mBio. 2019 Dec 10;10(6):e02759-19. doi: 10.1128/mBio.02759-19 (PMC6904878; doi:10.1128/mBio.02759-19)
Supplement: TABLE S2 [file mBio.02759-19-st002.pdf]

Table S2. Construction of *Y. pestis* Yop deletion strains and the oligos used for lambda red recombination to generate the strains used in this study.

Strain number	Strain name	Genotype	Background strain	Gene or region deleted	Oligos used for lambda red recombination
YP487-1	*Y. pestis ΔyopE/yopH/yopJ/ypkA*	*ΔyopE ΔKO1 (yopH, ypkA, yopJ)*	YP266-1 (*ΔyopE*)	KO1 (yopH, ypkA, yopJ)	n/a - strain KE52 used for homologous recombination
YP489	*Y. pestis Δyop*	*ΔyopE ΔKO1 (yopH, ypkA, yopJ), ΔKO2 (yopT, yopM, yopQ)*	YP487-1 (ΔyopE/yopH/yopJ/ypkA)	KO2 (yopT, yopM, yopQ)	BG2977, BG2989, BG2990, BG2978
YP493	*Y. pestis ΔyopE/yopH*	*ΔyopE ΔyopH*	YP266-1 (*ΔyopE*)	yopH	BG3011, BG3012, BG3013, BG3014
YP500-2	*Y. pestis ΔyopE/ypkA*	*ΔyopE ΔypkA*	YP266-1 (*ΔyopE*)	ypkA	BG3043, BG3044, BG3045, BG3006
YP522	*Y. pestis ΔypkA/yopJ*	*ΔypkA-yopJ*	YP03.5 (wild-type)	ypkA-yopJ	BG3006, BG3065, BG3021, BG3022
YP525	*Y. pestis ΔyopH/ypkA*	*ΔyopH ΔypkA*	YP373-1 (*ΔyopH*)	ypkA	BG3043, BG3044, BG3045, BG3006
YP526	*Y. pestis ΔyopH/yopJ*	*ΔyopH ΔyopJ*	YP373-1 (*ΔyopH*)	yopJ	BG3019, BG3020, BG3021, BG3022
YP528	*Y. pestis ΔyopT/yopM/yopQ*	*ΔKO2 (yopT, yopM, yopQ)*	YP03.5 (wild-type)	KO2 (yopT, yopM, yopQ)	BG2977, BG2989, BG2990, BG2978
YP529	*Y. pestis ΔyopE/yopJ*	*ΔyopE ΔyopJ*	YP100 (*ΔyopJ*)	yopE	BG2930, BG2931, BG2932, BG2933
YP520	*Y. pestis* yopE only	*ΔKO1 (yopH, ypkA, yopJ) ΔKO2 (yopT, yopM, yopQ)*	YP03.5 (wild-type)	(1) KO1 (yopH, ypkA, yopJ)<br>(2) KO2 (yopT, yopM, yopQ)	(1) n/a - strain KE52 used for homologous recombination<br>(2) BG2977, BG2989, BG2990, BG2978
YP517-1	*Y. pestis* yopH only	*ΔyopE ΔKO2 (yopT, yopM, yopQ) ypkA-yopJ::kan*	YP266-1 (*ΔyopE*)	(1) KO2 (yopT, yopM, yopQ)<br>(2) ypkA-yopJ	(1) BG2977, BG2989, BG2990, BG2978<br>(2) BG3006, BG3065, BG3021, BG3022
YP533-2	*Y. pestis* yopEH only	*ΔKO2 (yopT, yopM, yopQ) ΔypkA-yopJ*	YP522 (*ΔypkA-yopJ*)	KO2 (yopT, yopM, yopQ)	BG2977, BG2989, BG2990, BG2978
